# Impact of grouping complications on mortality in traumatic brain injury: A nationwide population-based study

**DOI:** 10.1371/journal.pone.0190683

**Published:** 2018-01-11

**Authors:** Chung-Han Ho, Fu-Wen Liang, Jhi-Joung Wang, Chung-Ching Chio, Jinn-Rung Kuo

**Affiliations:** 1 Department of Medical Research, Chi-Mei Medical Center, Tainan, Taiwan; 2 Department of Hospital and Health Care Administration, Chia Nan University of Pharmacy and Science, Tainan, Taiwan; 3 National Cheng Kung University Research Center for Health Data and Department of Public Health, College of Medicine, National Cheng Kung University, Tainan, Taiwan; 4 Department of Neurosurgery, Chi-Mei Medical Center, Tainan, Taiwan; 5 Department of Biotechnology, Southern Taiwan University of Science and Technology, Tainan, Taiwan; Hungarian Academy of Sciences, HUNGARY

## Abstract

Traumatic brain injury (TBI) is an important health issue with high mortality. Various complications of physiological and cognitive impairment may result in disability or death after TBI. Grouping of these complications could be treated as integrated post-TBI syndromes. To improve risk estimation, grouping TBI complications should be investigated, to better predict TBI mortality. This study aimed to estimate mortality risk based on grouping of complications among TBI patients. Taiwan's National Health Insurance Research Database was used in this study. TBI was defined according to the International Classification of Diseases, Ninth Revision, Clinical Modification codes: 801–804 and 850–854. The association rule data mining method was used to analyze coexisting complications after TBI. The mortality risk of post-TBI complication sets with the potential risk factors was estimated using Cox regression. A total 139,254 TBI patients were enrolled in this study. Intracerebral hemorrhage was the most common complication among TBI patients. After frequent item set mining, the most common post-TBI grouping of complications comprised pneumonia caused by acute respiratory failure (ARF) and urinary tract infection, with mortality risk 1.55 (95% C.I.: 1.51–1.60), compared with those without the selected combinations. TBI patients with the combined combinations have high mortality risk, especially those aged <20 years with septicemia, pneumonia, and ARF (HR: 4.95, 95% C.I.: 3.55–6.88). We used post-TBI complication sets to estimate mortality risk among TBI patients. According to the combinations determined by mining, especially the combination of septicemia with pneumonia and ARF, TBI patients have a 1.73-fold increased mortality risk, after controlling for potential demographic and clinical confounders. TBI patients aged<20 years with each combination of complications also have increased mortality risk. These results could provide physicians and caregivers with important information to increase their awareness about sequences of clinical syndromes among TBI patients, to prevent possible deaths among these patients.

## Introduction

Traumatic brain injury (TBI), a major cause of morbidity and mortality in many countries [1,2], is an important global public health problem. TBI is known as a silent disease because its complications are often associated with changes in thinking, feeling, language, or mood [3,4].

Patients with TBI commonly have many sequelae, such as sleep disorders [5], anxiety or depression [6], or post-traumatic stress disorder [7]. Studies have shown that these complications may lead to illness or death [8,9]; however, patients with TBI often pay little attention to these complications, as many of them are not obvious to patients or caregivers. In addition, many studies have suggested that TBI may cause or accelerate the progression of different diseases [9], such as stroke [10,11] and pneumonia [12].

The mortality risk owing to a single sequela after TBI has been analyzed in many studies, as mentioned above, and many researchers have developed different risk prediction models to estimate the probability of death in post-TBI patients [13–15]. However, a major challenge in evaluating the effect of multiple complications among patients with TBI is how to adequately and appropriately present complex postoperative complications among TBI patients. In particular, some complications after TBI are directly caused by brain injury and some are indirectly caused by other complications [9]. For example, stroke is a risk factor of death in all populations [16], and some studies have indicated that TBI is associated with a higher risk of stroke [10,11]. In addition, it has been shown that sleep problems are a common symptom during and after TBI [9,17,18] and that patients with sleep disorders have an increased risk of stroke. Yet it remains unknown how the combined effect of stroke and sleep disorders affects the risk of death among TBI patients. Therefore, diseases and symptoms should be grouped together as a more meaningful risk factor to examine the combined effect of disease and disease, symptoms and symptoms, or disease and symptoms on mortality risk in patients with TBI. The relationships between diseases and symptoms in large databases could be grouped using an association rule approach, to discover potential patterns [19].

To the best of our knowledge, few studies have attempted to explore the effect of grouping complications among post-TBI patients. Therefore, this study aimed not only to explore the associations among post-TBI complications but also to estimate mortality risk by grouping TBI complications in post-TBI patients, using a nationwide administrative database.

## Methods

### Data source

Taiwan launched a government-run single-payer National Health Insurance program in 1995. The medical claims database, the National Health Insurance Research Database (NHIRD), was established for research purposes. In this study, data were obtained from the NHIRD between January 1997 and December 2013. The NHIRD covers 99% of inpatient and outpatient medical benefit claims for Taiwan’s 23 million residents. The database comprises detailed information regarding clinical visits for each insured person, including date of diagnosis; diagnostic codes according to the International Classification of Diseases, Ninth Revision, Clinical Modification (ICD-9-CM); payments for consultations; and prescription details [20,21].

### Ethics statement

Encrypted personal identifiers are used in the NHIRD to protect patients’ privacy and prevent the possibility of an ethical violation according to regulations of the Bureau of National Health Insurance in Taiwan. The anonymous identification numbers have linked claims information, such as patient sex, date of birth, medical services received, and prescription information, which can be used by researchers. Therefore, informed consent was not required for the present study, which was approved for exemption by the Institutional Review Board (IRB) of Chi Mei Medical Center (IRB: 10410-E01).

### Patient selection and definition

Diagnostic codes were accessed via the inpatient and outpatient claims databases of the NHIRD. A total of 139,254 patients with TBI were enrolled in this study. TBI patients were defined as inpatients with a diagnosis code of TBI (ICD-9-CM codes 801–804 and 850–854), and who had intensive care unit (ICU) records between 2001 and 2012. We excluded patients who died within one month after TBI diagnosis owing to severe TBI. In addition, to ensure that all selected patients had new-onset TBI, we also excluded those with a TBI diagnosis before December 31, 2000.

Demographic variables (age and sex) and clinical characteristics, including length of stay (LOS), length of ICU stay, length of noninvasive ventilation (NV), and Charlson comorbidity index (CCI) score, were used as potential confounders to estimate the mortality risk among post-TBI patients. The CCI score is commonly used as a surrogate of patients’ disease severity [22,23]. Comorbidities measured by the CCI score are based on medical records for the one year prior to the date of TBI diagnosis; the CCI score has been used in many studies of TBI [24,25]. The length of ICU stay in TBI patients is often associated with a severe or poor clinical condition [26,27]. Therefore, the length of hospital stay, length of ICU stay, and the length of NV days among patients with TBI were further classified into four groups, according to their statistical distribution: a short-term group (minimum to first quartile), mild-term group (first quartile to median), long-term group (median to third quartile), and prolonged group (third quartile to maximum). This approach has been used in previous studies [28].

### Measurements

The main event in this study was mortality in post-TBI patients with more than 30 days’ survival. Follow-up time was calculated from the date of TBI admission to the date of death, withdrawal from the insurance system, or the end of the study (31 December, 2013), whichever came first. The last two conditions were defined as censors in this study. Mortality was defined as having a death record in the inpatient claim dataset; a ICD-9-CM diagnosis code of 798 (sudden death, cause unknown) in the emergency department, according to the outpatient claim dataset; or withdrawal from the insurance program and without reenrollment within 180 days of the last health care visit.

The main aim of this study was to examine the grouping of complications in post-TBI patients using the association rule method. Complications were based on ICD-9-CM diagnosis codes after the date of TBI admission, obtained from inpatient and outpatient claims datasets. However, only complications that occurred within the first year after the date of TBI admission were selected to estimate the mortality risk among post-TBI patients. The primary reason for selecting only complications within the first year was to avoid inclusion of diseases that may be unrelated to TBI, such as the common cold. In addition, we excluded patients with certain chronic diseases before TBI admission, such as hypertension (ICD-9-CM: 410), diabetes mellitus (DM) (ICD-9-CM: 250), and hyperlipidemia (ICD-9-CM: 272).

The association rule, a data mining approach, was used to evaluate multiple complications, to improve the accuracy of mortality risk prediction. The association rule method was first introduced by Piatetsky-Shaprio [19], and it is often used in large databases for pattern mining in different fields, such as computer science [29] and for medical applications [30,31]. In this study, frequent item set mining, an association rule method, was used to investigate the grouping of post-TBI complications for estimating mortality risk. The support (%) of complications set X was defined as the proportion of diagnoses among all health care visits after the date of TBI admission from inpatient and outpatient datasets. Support is a measure of how frequently the rule occurs in the database. The resultant integrated post-TBI risk profile will provide useful information for TBI patients and their families.

### Statistical analysis

The frequency with percentage and median with interquartile range (IQR) were presented for discrete variables and continuous variables, respectively. The Pearson’s chi-squared test was used to examine distribution differences by age group, sex, comorbidities, categorized CCI score, LOS, length of ICU stay, and length of NV days between patients with TBI who were alive and those who died. The Wilcoxon rank-sum test was used to compare LOS, length of ICU stay, and length of NV days at first TBI diagnosis between deceased and alive TBI patients.

In addition, the Kaplan-Meier failure plot was applied to describe the cumulative incidence rate of mortality, and the log-rank test was used to compare the risk difference between patients with grouping of complications and those without grouping. A Cox proportional regression model was used to estimate the mortality risk for different complication groupings, adjusted for potential confounding factors including age, sex, CCI score, LOS, length of ICU stay, and length of NV days. The proportional hazards assumption was assessed using Schoenfeld residuals for each variable of interest.

To test the hypothesis that the effect of grouping post-TBI complications on mortality varied by age and sex in patients with TBI, the model was also stratified by age and sex groups. SAS version 9.4 software (SAS Institute, Inc, Cary, NC, USA) was used to perform all statistical analyses. Kaplan-Meier curves were generated using Stata version 12 (StataCorp, College Station, TX, USA). All significance levels were set at a p-value <0.05.

## Results

A total of 139,254 patients were enrolled in this study. The distribution of the baseline characteristics between TBI patients who died before the end of the study and those who were still alive is presented in Table 1. The median follow-up time for the enrolled participants was 4.39 years (IQR = 1.76–7.63 years). The distribution of all characteristics, including age, sex, and comorbid conditions, was significantly different between the deceased group and the survival group. Compared with TBI patients who were still alive, those patients who died were significantly older and had higher CCI scores, longer lengths of stay, longer lengths of ICU stay, and a longer length of NV days. The median LOS for deceased TBI patients was 16 days (IQR = 8.0–30.0 days) and that for the survival group was 10 days (IQR = 6.0–17.0 days). The median length of ICU stay for TBI patients who died and those who survived was 5 days (IQR = 2.0–15.0 days) and 3 days (IQR = 1.0–5.0 days), respectively. Furthermore, as predicted, TBI patients who died before the end of study had more comorbid conditions. Among deceased TBI patients, 17.7% had cerebrovascular disease, 13.2% had chronic pulmonary disease, 12.3% had ulcer disease, and 23.8% had DM. Among TBI patients who survived, 5.0% of them had cerebrovascular disease, 4.2% had chronic pulmonary disease, 5.1% had ulcer disease, and 9.4% had DM. In addition, TBI patients aged over 65 years had the highest risk of death (HR = 27.64, 95% CI = 24.64–29.71). Male TBI patients had 1.14 times (95% CI = 1.11–1.17) greater risk for death than their female counterparts. Higher CCI scores were associated with higher risk of death; patients with a baseline CCI score of 2 had 4.59 times (95% CI = 4.42–4.78) greater risk of death and those with a baseline score of 3 had 6.69 times (95% CI = 6.49–6.70) greater risk, compared with patients with a baseline score of 0. For each one-day increase in length of ICU stay, there was a 5% (IQR = 2.00–15.00) increase in the mortality risk. Patients with one additional day of ventilator use had a 4% (95% CI = 1.043–1.045) increased risk of death.

**Table 1:** Baseline information of 139,254 patients with TBI from 2001–2012.

Variables	Died (N = 26,604)	Survived (N = 112,650)	p-value*	Crude hazard ratio (95% CI)
Age (years), n (%)				
<20	451 (1.70)	18659 (16.56)	< .0001	Ref.
20–34	1319 (4.96)	23205 (20.60)		2.40 (2.15–2.67)
35–49	2728 (10.25)	21106 (18.74)		5.33 (4.83–5.89)
50–64	4425 (16.63)	23061 (20.47)		8.62 (7.83–9.50)
≥65	17681 (66.46)	26619 (23.63)		27.64 (24.64–29.71)
Sex, n (%)				
Female	7933 (29.82)	38210 (33.92)	< .0001	Ref.
Male	18671 (70.18)	74440 (66.08)		1.14 (1.11–1.17)
Comorbidity, n (%)				
Myocardial infarction	363 (1.36)	441 (0.39)	< .0001	3.46 (3.12–3.83)
Congestive heart failure	1761 (6.62)	1500 (1.33)	< .0001	4.59 (4.37–4.82)
Peripheral vascular disease	352 (1.32)	425 (0.38)	< .0001	3.40 (3.06–3.77)
Cerebrovascular disease	4705 (17.69)	5642 (5.01)	< .0001	3.66 (3.55–3.78)
Dementia	2189 (8.23)	2366 (2.10)	< .0001	4.01 (3.84–4.19)
Chronic pulmonary disease	3506 (13.18)	4760 (4.23)	< .0001	2.99 (2.89–3.10)
Rheumatologic disease	167 (0.63)	411 (0.36)	< .0001	1.81 (1.55–2.11)
Ulcer disease	3278 (12.32)	5777 (5.13)	< .0001	2.40 (2.31–2.49)
Mild liver disease	1806 (6.79)	3216 (2.85)	< .0001	2.31 (2.20–2.42)
DM	6335 (23.81)	10538 (9.35)	< .0001	3.12 (3.03–3.21)
Hemiplegia	387 (1.45)	353 (0.31)	< .0001	3.47 (3.14–3.83)
Moderate or severe renal disease	2096 (7.88)	1947 (1.73)	< .0001	4.76 (4.55–4.98)
DM with end organ damage	1777 (6.68)	2241 (1.99)	< .0001	3.49 (3.33–3.67)
Any tumor	1869 (7.03)	2096 (1.86)	< .0001	4.08 (3.89–4.28)
Moderate or severe liver disease	410 (1.54)	317 (0.28)	< .0001	4.51 (4.09–4.980
Metastatic solid tumor	350 (1.32)	185 (0.16)	< .0001	7.21 (6.49–8.01)
AIDS	—	—	-	-
CCI score classification, n (%)				
0	11453 (43.05)	86876 (77.12)	< .0001	Ref.
1	5440 (20.45)	13570 (12.05)		3.03 (2.94–3.13)
2	3119 (11.72)	4976 (4.42)		4.59 (4.42–4.78)
3	6592 (24.78)	7228 (6.42)		6.69 (6.49–6.70)
LOS (days), median (IQR)	16.00 (8.00–30.00)	10.00 (6.00–17.00)	< .0001	1.02 (1.019–1.020)
LOS (days)–groups, n (%)				
<6 (≤Q1)	4949 (18.60)	32659 (28.99)	< .0001	Ref.
6–11 (Q1–Q2)	5093 (19.14)	30913 (27.44)		1.08 (1.04–1.13)
11–20 (Q2–Q3)	5794 (21.78)	26922 (23.90)		1.36 (1.31–1.41)
<20 (>Q3)	10768 (40.48)	22156 (19.67)		2.80 (2.71–2.90)
ICU stay (days), median (IQR)	5.00 (2.00–15.00)	3.00 (1.00–5.00)	< .0001	1.05 (1.052–1.054)
ICU stay (days)–groups, n (%)				
0–2 (≤Q1)	8306 (31.22)	55449 (49.22)	< .0001	Ref.
2–3 (Q1–Q2)	2893 (10.87)	15645 (13.89)		1.23 (1.18–1.29)
3–6 (Q2–Q3)	3962 (14.89)	18970 (16.84)		1.36 (1.31–1.41)
<6 (>Q3)	11443 (43.01)	22586 (20.05)		3.05 (2.97–3.14)
Ventilation (days), median (IQR)	10.00 (3.00–20.00)	3.00 (1.00–8.00)	< .0001	1.04 (1.043–1.045)
Ventilation (days)–groups, n (%)				
0–2 (≤Q1)	3079 (21.93)	18611 (42.97)	< .0001	Ref.
2–4 (Q1–Q2)	1450 (10.33)	6738 (15.56)		1.30 (1.22–1.38)
4–11 (Q2–Q3)	3064 (21.83)	10342 (23.88)		1.79 (1.71–1.89)
<11 (>Q3)	6444 (45.91)	7622 (17.60)		4.52 (4.33–4.72)

* p-values were calculated using the Pearson’s chi-squared test for categorical variables and Wilcoxon rank-sum test for continuous variables.

Abbreviations: TBI, traumatic brain injury; CI, confidence interval; DM, diabetes mellitus; LOS, length of stay; IQR, interquartile range; Q1, first quartile; Q2, second quartile; Q3, third quartile; ICU, intensive care unit.

The five most common complications occurring within one year of TBI diagnosis are shown in Table 2. A total 3,853,713 diagnostic records of post-TBI patients within one year were included in this study. Intracerebral hemorrhage (2.66%) was the most common complication within the first year after TBI, followed by acute upper respiratory infections of multiple or unspecified sites (1.75%), dizziness and giddiness (1.21%), constipation (1.03%), and urinary tract infection (0.92%).

**Table 2:** Top five complications within one year among post-TBI patients.

Rank	ICD-9-CM diagnostic codes	Frequency	%
1	**431** Intracerebral hemorrhage	195,840	2.66
2	**465.9** Acute upper respiratory infections of multiple or unspecified sites	128,439	1.75
3	**780.4** Dizziness and giddiness	88,655	1.21
4	**564.0** Constipation	76,030	1.03
5	**599.0** Urinary tract infection, site not specified	67,756	0.92

Note: Total number of diagnostic records: 3,853,713.

Abbreviations: TBI, traumatic brain injury; ICD-9-CM, International Classification of Diseases, Ninth Revision, Clinical Modification.

Most patients included in this study had more than one complication. Using the frequent item set mining approach, we found five sets of the most common post-TBI grouping combinations that were highly correlated (Table 3). The most common set was pneumonia with acute respiratory failure (ARF) and urinary tract infection (UTI) (N = 16,859, 0.055%), followed by the set of unspecified septicemia with pneumonia and ARF (N = 12,988, 0.042%), the set of unspecified septicemia with ARF and UTI (N = 9,778, 0.032%), the set of unspecified septicemia with pneumonia and UTI (N = 8,423, 0.027%), and the combination of pneumonia with chronic respiratory failure and UTI (N = 6,535, 0.021%).

**Table 3:** Top five grouped complications within the first year after TBI.

Rank	Combined complications (ICD-9-CM)	Frequency	%
1	Combination 1: **486** Pneumonia, organism unspecified **518.81** Acute respiratory failure **599.0** Urinary tract infection, site not specified	16,859	0.055
2	Combination 2: **038.9** Unspecified septicemia **486** Pneumonia, organism unspecified **518.81** Acute respiratory failure	12,988	0.042
3	Combination 3: **038.9** Unspecified septicemia **518.81** Acute respiratory failure **599.0** Urinary tract infection, site not specified	9,778	0.032
4	Combination 4: **038.9** Unspecified septicemia **486** Pneumonia, organism unspecified **599.0** Urinary tract infection, site not specified	8,423	0.027
5	Combination 5: **486** Pneumonia, organism unspecified **518.83** Chronic respiratory failure **599.0** Urinary tract infection, site not specified	6,535	0.021

Abbreviations: TBI, traumatic brain injury; ICD-9-CM, International Classification of Diseases, Ninth Revision, Clinical Modification.

**Fig 1:**
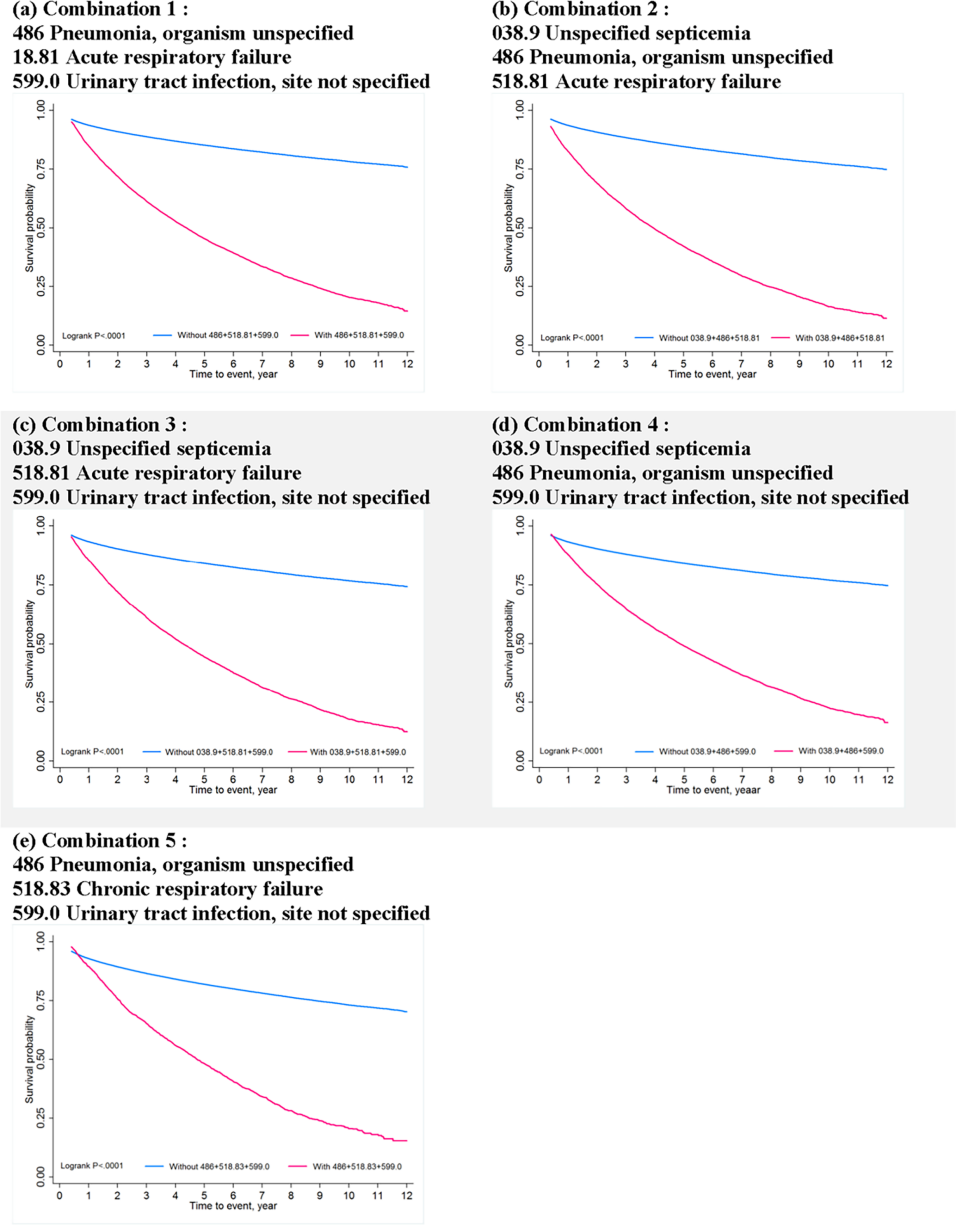
Kaplan-Meier failure estimates of mortality between TBI patients with and without grouped post-TBI complications.

Kaplan-Meier failure estimates of mortality between TBI patients with and without grouping of post-TBI complications are shown in Fig 1. Consistently, the survival probability for TBI patients with any set of grouped complications was significantly lower than those without grouped complications (log-rank test, p < .0001) over the follow-up period.

In the Cox regression model (Table 4), each combination of post-TBI complications was a significant predictor of mortality. After adjusting for age, sex, CCI score, LOS, length of ICU stay, and length of ventilation days, most sets of grouped complications were significantly associated with increased risk of death, except for the set comprising pneumonia, chronic respiratory failure, and UTI.

**Table 4:** Hazard ratios of mortality among TBI patients with grouping of post-TBI complications, compared with those without grouping.

Complication groupings	Crude HR (95% CI)	p-value	Adjusted HR (95% CI)	p-value
Combination 1: **486** Pneumonia, organism unspecified **518.81** Acute respiratory failure **599.0** Urinary tract infection, site not specified	4.89 (4.76–5.02)	< .0001	1.55 (1.51–1.60)	< .0001
Combination 2: **038.9** Unspecified septicemia **486** Pneumonia, organism unspecified **518.81** Acute respiratory failure	5.20 (5.06–5.35)	< .0001	1.73 (1.68–1.78)	< .0001
Combination 3: **038.9** Unspecified septicemia **518.81** Acute respiratory failure **599.0** Urinary tract infection, site not specified	4.72 (4.59–4.86)	< .0001	1.53 (1.48–1.58)	< .0001
Combination 4: **038.9** Unspecified septicemia **486** Pneumonia, organism unspecified **599.0** Urinary tract infection, site not specified	4.20 (4.08–4.32)	< .0001	1.38 (1.34–1.42)	< .0001
Combination 5: **486** Pneumonia, organism unspecified **518.83** Chronic respiratory failure **599.0** Urinary tract infection, site not specified	3.62 (3.45–3.80)	< .0001	1.03 (0.97–1.08)	0.3504

Abbreviations: TBI, traumatic brain injury; HR, hazard ratio; CI, confidence interval.

The results of stratified analyses by age and sex are presented in Table 5. For each age group, after controlling for potential confounders, the grouping of complications was associated with increased risk of death, and the effect was stronger in younger patients with Combinations 2, 3, and 4. However, elderly TBI patients (aged 65 years or more) with Combination 5 had a significantly lower risk of death compared with those of the same age who did not have that set of complications (HR = 0.92, 95% CI = 0.87–0.98). In addition, grouping of complications increased the risk of death in both men and women, and the adverse effect was stronger in female TBI patients.

**Table 5:** Hazard ratios of mortality among TBI patients, with and without combinations of three complications, stratified by age and sex.

Variable	Combination 1		Combination 2		Combination 3		Combination 4		Combination 5	
	Adjusted HR (95% CI)	p-value	Adjusted HR (95% CI)	p-value	Adjusted HR (95% CI)	p-value	Adjusted HR (95% CI)	p-value	Adjusted HR (95% CI)	p-value
Age (years)										
<20	1.91 (1.34–2.72)	0.0003	4.95 (3.55–6.88)	< .0001	3.49 (2.36–5.16)	< .0001	2.91 (1.97–4.32)	< .0001	3.72 (2.31–5.99)	< .0001
20–34	2.77 (2.26–3.40)	< .0001	3.86 (3.13–4.75)	< .0001	2.74 (2.15–3.48)	< .0001	2.56 (2.02–3.24)	< .0001	2.82 (2.05–3.87)	< .0001
35–49	2.55 (2.27–2.87)	< .0001	2.89 (2.57–3.25)	< .0001	2.70 (2.38–3.05)	< .0001	2.33 (2.05–2.63)	< .0001	1.98 (1.65–2.39)	< .0001
50–64	2.06 (1.90–2.22)	< .0001	2.43 (2.25–2.62)	< .0001	2.07 (1.91–2.25)	< .0001	1.87 (1.73–2.02)	< .0001	1.38 (1.21–1.57)	< .0001
≥65	1.42 (1.37–1.46)	< .0001	1.54 (1.49–1.60)	< .0001	1.39 (1.34–1.44)	< .0001	1.25 (1.21–1.30)	< .0001	0.92 (0.87–0.98)	0.0073
Sex										
Male	1.52 (1.47–1.58)	< .0001	1.72 (1.66–1.79)	< .0001	1.47 (1.41–1.52)	< .0001	1.35 (1.30–1.40)	< .0001	1.00 (0.94–1.06)	0.9715
Female	1.63 (1.54–1.72)	< .0001	1.76 (1.66–1.86)	< .0001	1.69 (1.60–1.78)	< .0001	1.45 (1.38–1.54)	< .0001	1.10 (1.00–1.21)	0.0473

Note: Adjusted variables were Charlson Comorbidity Index score, length of stay, days in intensive care unit, and length of NV days.

## Discussion

To the best of our knowledge, this is the first study to report the risk of mortality based on grouping of post-TBI complications among TBI patients using population-based administrative data. The main findings of this study were as follows. (1) The five most common post-TBI complications were related to a clinical diagnosis of TBI; these were intracerebral hemorrhage, acute upper respiratory infections, dizziness and giddiness, constipation, and urinary tract infection. (2) The main combinations of post-TBI complications were commonly found in respiratory care centers or respiratory care wards; these included pneumonia, acute respiratory failure, urinary tract infection, unspecified septicemia, and chronic respiratory failure. (3) The adverse effects were greater when the grouping of TBI complications included unspecified septicemia. (4) The effect of increased mortality risk owing to grouping of post-TBI complications was greater in younger and in female TBI patients.

Consistent with our previous studies, older age, male sex, higher CCI scores, longer length of hospital stay, longer ICU stay, longer ventilator use, and comorbidities of DM or cerebrovascular, cardiac, renal, or liver diseases were associated with significantly higher mortality (Table 1) [32,33]. Our results showed that intracerebral hemorrhage (2.66%), a type of cerebrovascular accident, was the most common diagnosis among post-TBI patients (Table 2). Intracerebral hemorrhage is one of the diagnosis codes used in the definition of stroke in previous studies [34]. Stroke is the second leading cause of death worldwide, and it is also the main cause of disability in adults [35]. In addition, most studies have indicated that TBI patients have a higher risk of stroke [11,36]. Hence, the results of the present study provide powerful evidence for the high incidence of intracerebral hemorrhage among post-TBI patients.

Recently, co-existence of two or more chronic diseases in an individual, also known as multiple morbidity, complex morbidity, or complex chronic disease, has been proved to have a much greater impact on health care systems than previously thought [37], such as by reducing life quality and life expectancy [38,39] and increasing drug–drug interaction side effects [40]. However, when discussing the effects of complications on mortality, potential prognostic factors are usually evaluated individually. The mortality risk based on grouping of post-TBI complications has not been investigated. In particular, most studies of TBI have used the neural network approach, a machine learning method, to predict potential risk factors [41–43]; however, grouping complications using association rule mining may offer a novel approach to evaluating the combined effects of risks in the real world. Therefore, this issue is worth further investigation, as our results have shown.

Our findings indicated that the most common set of post-TBI complications was pneumonia with acute respiratory failure (ARF) and urinary tract infection (UTI) (0.055%); other combinations were also highly related to pneumonia. Patients with TBI have been shown to have a greater risk of pneumonia [44,45]. In addition, TBI patients might have airway obstruction, aspiration, or hypoxia [46], which could lead to pneumonia, as could using intubation or mechanical ventilation [47]. Our findings when grouping complications were consistent with those other studies, as pneumonia, ARF, and UTI are well-known post-TBI complications [48].

Septicemia, a common post-TBI complication, might be associated with other neurological outcomes, according to different levels of disease severity [49]. Studies with rat models have indicated that sepsis would increase the post-TBI mortality rate [50,51]. In our study, Combinations 2 and 3, which include septicemia, presented higher mortality risk (Combination 2: HR = 1.73, 95% CI = 1.68–1.78; Combination 3: HR = 1.53, 95% CI = 1.48–1.58) than other groupings. Therefore, it is important that intensive chest care, a timely fever workup, and adequate antibiotic treatment are provided for patients with TBI, to prevent septicemia.

Moreover, the risk of death among TBI patients according to age and sex varied, as the prognosis differed according to these factors [52,53]. Age is directly associated with post-TBI mortality [54,55]. Thus, a stratified analysis by different ages and sex was necessary. In our study, the effect of increased mortality risk was stronger among younger TBI patients. A possible reason for this effect is that younger adults might receive lower levels of critical care consultation, which may lead to higher mortality in this population [56]. This implies that TBI patients who are younger or female and who have the identified grouping combinations are more vulnerable (Table 5). Therefore, we emphasize that all health caregivers in daily practice should be alert when dealing with high-risk TBI patients who have lung or urinary system illnesses, to prevent the occurrence of pneumonia, respiratory failure, urinary tract infection, and septicemia after TBI.

Some limitations should be noted. First, all diagnoses were based on claims data and ICD-9-CM diagnosis codes, which might result in disease misclassifications, missing data, or reporting discrepancies. However, all diagnostic records were coded by medical professionals, and many studies have validated the accuracy of the main diagnosis codes in the NHIRD [57–60]. Second, the level of disease severity, imaging information, and behavioral information such as smoking or alcohol consumption, were unavailable in the database. Although the CCI score, the length of ICU stay, and the length of NV days were used to estimate the severity level among TBI patients, validation using a clinical severity scale such as the Glasgow Coma scale or Barthel scale, as well as the severity variables mentioned above, should be considered in future research. In addition, the causes of TBI such as vehicular accidents or falls, which might be related to disease severity, were unknown in our study. Finally, although we excluded patients who died within one month after TBI diagnosis, multiple injuries may also affect the mortality risk of patients as they might have higher levels of clinical severity.

In conclusion, we used population-based health insurance claims data to demonstrate that grouping of post-TBI complications was associated with increased mortality risk. The findings of this study could improve the limitation of using a single disease to estimate mortality risk in future TBI research. These results can provide physicians and caregivers with important information to increase awareness about certain combinations of clinical syndromes among TBI patients.
